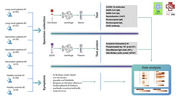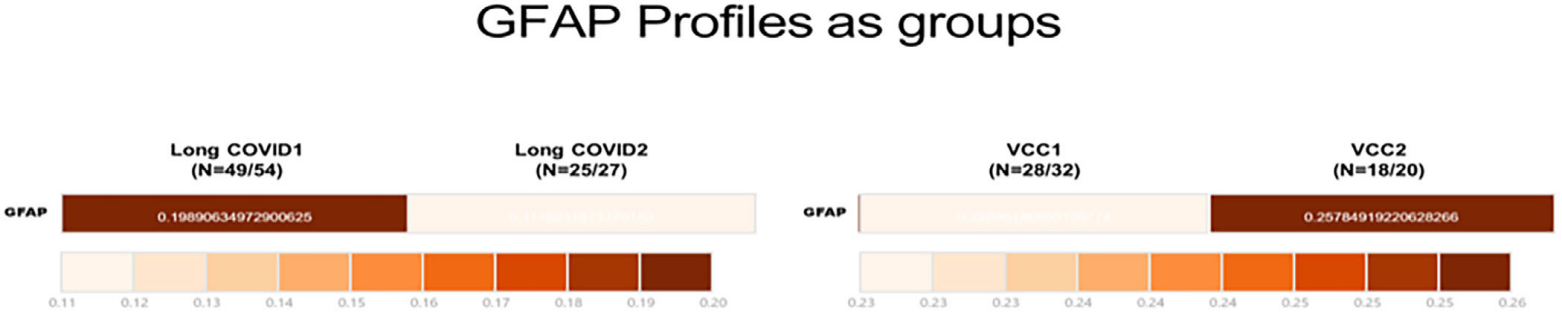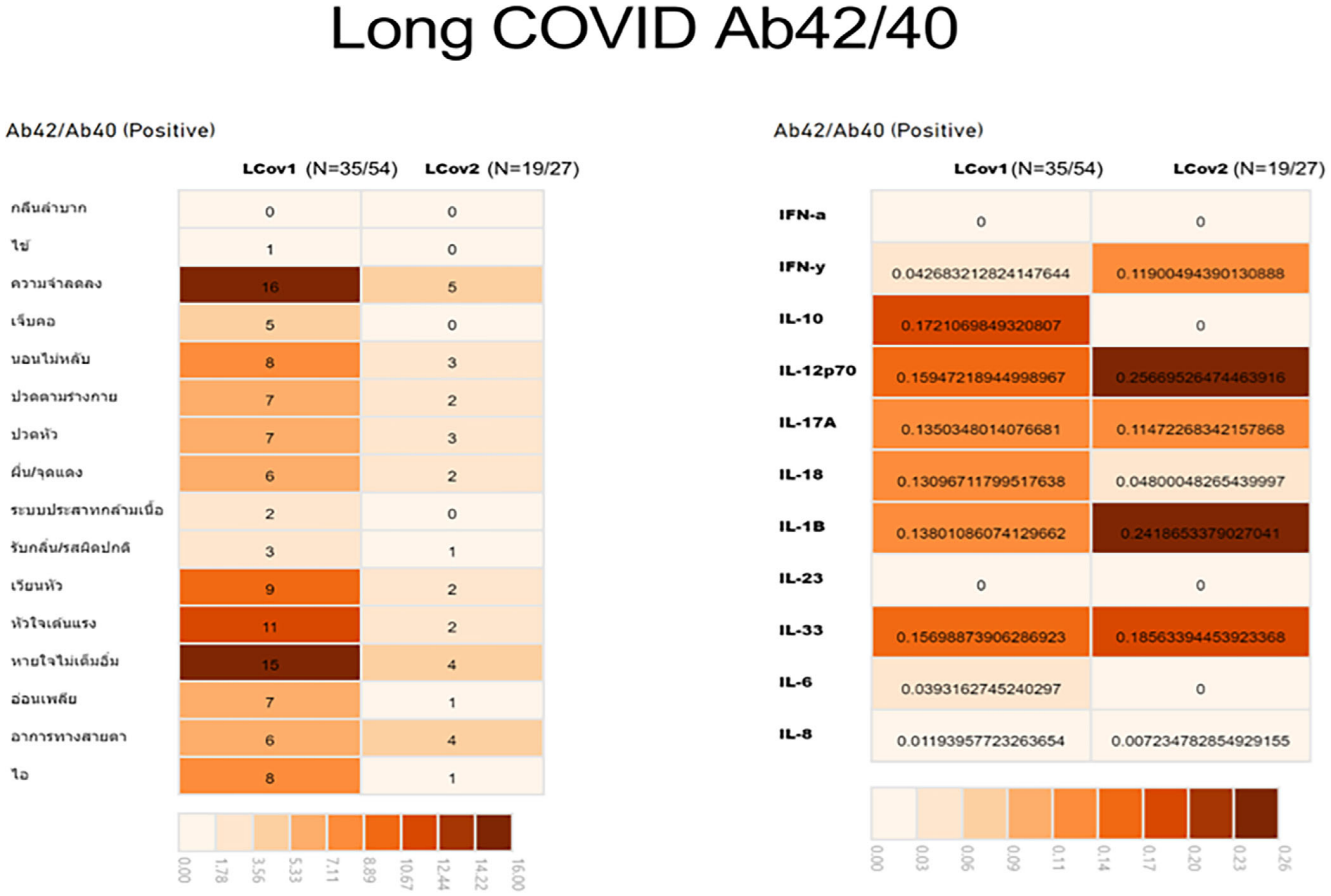# Development of Alzheimer’s Disease‐Related Blood Biomarker in Long COVID Patient

**DOI:** 10.1002/alz.094892

**Published:** 2025-01-09

**Authors:** Chanida Ruchisrisarod

**Affiliations:** ^1^ Thai Red Cross Emerging Infectious Diseases Health Science Centre, King Chulalongkorn Memorial Hospital, Bangkok Thailand

## Abstract

**Background:**

The COVID‐19 pandemic has a significant global health impact. A substantial number of infected individuals continue to experience post‐acute sequelae that affect daily life activities. This study aims to assess blood biomarkers for Alzheimer’s disease, inflammatory markers, and antibody indices related to various organ symptoms, particularly neuropsychiatric and other pro‐inflammatory conditions, in Long COVID patients.

**Methods:**

Patients with symptoms as defined in criteria regardless were recruited in this study. Assessment on clinical conditions was done. Blood samples were collected for analysis of antibodies to COVID. Cytokines assay and Assay for accumulation of abnormal beta amyloid as ratio between 42/40, p‐Tau 181 and brain damage markers (NFL neuro filament light chain and GFAP glial fibrillary acidic protein) was also done in parallel. Data analysis performed in correlation with clinical symptoms.

**Results:**

One hundred and seventy‐five individuals. All antibody panels were similar in pattern except antibody to N protein, indicative of infection found almost exclusively in patient. Antibody to ACE 2 was found in approximately 10% and much diminished thereafter suggesting a nonsignificant role in contributing prolonged symptoms. Cytokine profiling determined as individual group and between groups of all visits followed roughly similar pattern in both groups with markedly elevated IL‐1b, gamma interferon, IL‐12p70 and IL‐33. Cytokine responses in patients who had amyloid positive were gamma interferon IL 33, IL10 IL 17a and IL 18, IL10, IL 18 and IL12p70 in Tau positive IL 10, IL 18, IL33, IL6 and IL12p70 in NFL positive. GFAP was prominent in patient. This suggests that brain damaging process may not entirely resolved since astrocyte activation (GFAP positive) leads to amyloid induction and Tau pathology albeit disappearance of all symptoms. This is also true in cytokines response that remained elevated.

**Conclusions:**

This knowledge can be applicable not only to the current pandemic but also to future ones. Significant associations between specific neurological biomarkers and long COVID symptoms have been observed. Further studies can attempt to identify neurological biomarkers in the blood and utilize them as diagnostic and management tools for long COVID patients in clinical practice.